# A tale of two rivers: development, destruction, and despair in Ongata Rongai, Kenya

**DOI:** 10.3389/fpubh.2023.1164881

**Published:** 2023-08-25

**Authors:** Olivia Howland

**Affiliations:** ^1^Institute for Infection, Veterinary and Ecological Sciences (IVES), University of Liverpool, Liverpool, United Kingdom; ^2^International Livestock Research Institute, Nairobi, Kenya

**Keywords:** urban rivers, rapid growth, rural urban migration, wash, riparian, liminal space, human wildlife interactions, multispecies interactions

## Abstract

**Introduction:**

Ongata Rongai is a rapidly growing peri-urban space in Nairobi Metropolitan, Kenya. The last 10 years have seen exponential population growth and building development leading to overcrowding and pressure on water and environmental resources. This original research sheds light on interactions among humans, animals, and this rapidly changing urban environment. It is therefore a quintessentially One Health study.

**Methods:**

Qualitative and ethnographically informed methods are employed to better understand the impact of rapid growth on the riparian environment and the effect of this on those who depend on it. The reflexive use of archival material and a historical ethnographic approach enabled in-depth narratives to address these issues within a longitudinal context, and the use of deliberate walking alongside visual methodologies and more traditional anthropological methods make this study novel both in terms of methodological approach and findings.

**Results:**

This study finds that people cite high levels of pollution from solid waste and sewage have made the rivers almost unusable and a hazardous place for both humans and animals. Yet, in the past, these rivers played key roles in daily life. There is frustration with structural-level actors' moribund attitude to the environment. The poor health of the rivers and riparian environment leads to human and animal health challenges, increased pressure on water resources, and economic pressure due to a loss of livelihoods.

**Discussion:**

The study contributes to what is currently a fairly small literature on urban riparian spaces globally, but one which is of growing and vital importance given the rapidly increasing percentage of humans who now reside in urban spaces. It contributes to WASH and urban clean water knowledge as well as One Health, public health, and urban growth narratives, and directly addresses challenges faced by SDG 6.

## 1. Introduction

It is predicted that by 2050, seven out of 10 will live in cities and urban spaces (1). SDG^1^ 6 (2), which concerns clean water and sanitation, is one of the biggest challenges for urban spaces and those who live there. Waterborne diseases cause a high burden, with diarrheal disease still the 5th most common cause of death in low-income countries and the 6th in low- and middle-income countries (3). Exponential population growth adds pressure to water resources (4) which already suffer from the effects of climate change (5). Among climate-related issues are flooding, drought, contamination of water sources, and increased demand including extraction of water for domestic and commercial uses (5). Increased pressure on water resources leads to contamination which results in potentially fatal illnesses (4). Urban water security is vital to human, animal, and environmental health (6). This trifecta of actors, human, animal, and environmental, and the idea that they are intrinsically linked in a system of health, is called One Health (7).

Urban rivers are known to be places where anthropogenic activities play a role in rapid degradation (8, 9). Many of these activities cause pollution, rendering the water unusable for this and future generations (10–13) as well as significant health risks for humans and animals (14, 15). Multispecies interactions occur in urban riparian spaces (16), yet for many, the level of degradation makes rivers a no-go zone (17), enforcing this idea that rivers are *liminal* spaces. This term, first used in this context by anthropologist Victor Turner, has come to mean something which is not one thing nor another, a marginal place, a place in-between, a ritual state of becoming or unbecoming, or of being on the threshold of a new identity or status. Here, I use the term to mean the way in which rivers are boundaries, not one side nor another, and indeed the idea that they give and take away, are simultaneously clean and unclean, and safe and unsafe. Rivers are constantly changing, and it is this idea of flux which I believe makes rivers singularly liminal in their being.

Liminality can make some fearful of these spaces, yet others make use of this liminality: the appropriation of marginal spaces by youths has been well-documented (18). Riparian spaces host important plants for medicines (19) but also act as a place to dispose of trash and sewage (20).

Despite this, few researchers have ventured into these liminal African riparian spaces. This study sheds light on this emerging and vital area of research, which has far-reaching implications for One Health, multispecies interactions, and urban human and environmental futures. This study uses a mixed methods ethnographic approach to present the story of two rivers in a rapidly growing urban community in Nairobi, Kenya. These rivers are important as they are not in any way particular: they are just two of many rivers in the Nairobi River Basin and can perhaps be seen as a snapshot of a broader problem, that of urban rivers in LAMIC settings globally.

Here, I combine a novel set of methods including walking, archival research, interviews, photography, and reflexive techniques, to illuminate neglected urban riparian spaces and the realities of living and working in or near them.

The goal of this study is 2-fold: first, and perhaps most importantly, I present to you the historical and contemporary narratives of the rivers of Ongata Rongai, and second, I will demonstrate that a mix of creative methodologies is uniquely useful to gather these embedded and longitudinal data and to add to the growing body of evidence of the dangers and potential value of urban riparian spaces.

To achieve this, I use a Complexity and Systems Theoretical approach (21), which in turn feeds into a One Health framework: that is, all aspects of One Health (people, animals, and the environment) are inextricably linked within a system, and each aspect of this system affects the other/s. For example, if people are releasing untreated sewage into the river, then this will negatively affect the health and wellbeing of livestock, fish in the river, the environment, and in turn other humans who might consume the water and contract diarrheal diseases, or they might ingest antimicrobial resistant bacteria. Antibiotic-resistant pathogens have been found in water globally, and rivers are considered to be a hub for infection. Studies have found these pathogens persist in the environment including in river sediment and wildlife even after areas have been decontaminated (22). However, this theoretical framework has been accused of being reductionist, so I combine this thinking with a Complex Adaptive Systems approach which perhaps allows for a greater depth of intersectionality and understanding of the impact of aspects such as class and gender. This research was born out of a general understanding of One Health and riparian importance in epidemiology but takes an ethnographic approach in both methodology and analysis.

This study is significant because the current body of knowledge on urban riparian environments is lacking, and yet the importance of these spaces for human, animal, and environmental health cannot be overstated in a rapidly urbanizing world. This study is uniquely impactful as it uses local voices and embedded narratives of “experts”—those who have lived their entire lives in Ongata Rongai—to draw out the problems and issues specific to these people at this time, as well as shedding light on longitudinal change, to communicate the importance of a focus on the environmental aspect of One Health which is often neglected.

## 2. Materials and methods

### 2.1. Context

#### 2.1.1. Study site

The study site is Ongata Rongai and the two rivers which form the boundaries to this area: the Empakasi, Embakasi, or Mbagathi River (the spelling and pronunciation differ by tribe) and the Kiserian River which merges with the Kandisi River. These two rivers form the boundary between Nairobi County and Kajiado County, Ongata Rongai and the Nairobi National Park, and contain much of what was the “original” settlement of Ongata Rongai.

Ongata Rongai is a rapidly growing peri-urban settlement with a range of demographics which I believe is fairly indicative and representative of the Nairobi Metropolitan area as a whole. It was chosen as the study site because I have been a resident of the area for several years and have seen the progressive degeneration of these urban rivers, as well as the value which they bring to a range of stakeholders. This dichotomy, alongside the paucity of existing data on urban rivers in Kenya, demanded an investigation.

Historically, Ongata Rongai (from the Maa, meaning a narrow plain, i.e., the narrow plain bounded by the rivers) was a Maasai area, sparsely populated, mostly savannah grasslands with few permanent *bomas* or Maasai homesteads. Indeed, maps and aerial photographs obtained from several sources show little to no permanent structures in the area. It was an area very much peripheral to Nairobi city, yet today, there is no visual boundary between the two as urban sprawl has eaten up any vacant land or bush which might have existed between the two. Ongata Rongai, or Rongai,^2^ is characterized by poorly constructed, low-quality high-rise blocks of apartments, a prevalence of litter for which disposal services are sorely lacking, smoke from piles of burning rubbish, an often overpowering smell of rotting organic material from the market which spills out onto the road, and the smell of sewage from open drains and culverts.

Rongai contains multitudes and dichotomies: it also has some of the most expensive property in the country, with mansions belonging to government officials, expats, and wealthy Kenyans bordering the National Park along Maasai Lodge Road. Yet, Kware, an area of informal housing and high population density, is at the core of Rongai. The riparian areas are an interesting intersection of rich and poor, and beauty and horrors. Areas such as Rimpa, Kandisi, and Nkoroi are favored by middle-class Kenyans who have bought small plots and built elaborate houses, whereas Olekasasi and Tumaini are favored by more working-class people and students living in small rented apartments and single rooms. Rongai is diverse and could be considered a microcosm for the wider Nairobi Metropolitan area, with a large proportion of commuters since it is still more affordable than areas closer to the Nairobi city center.

**Figure 1:**
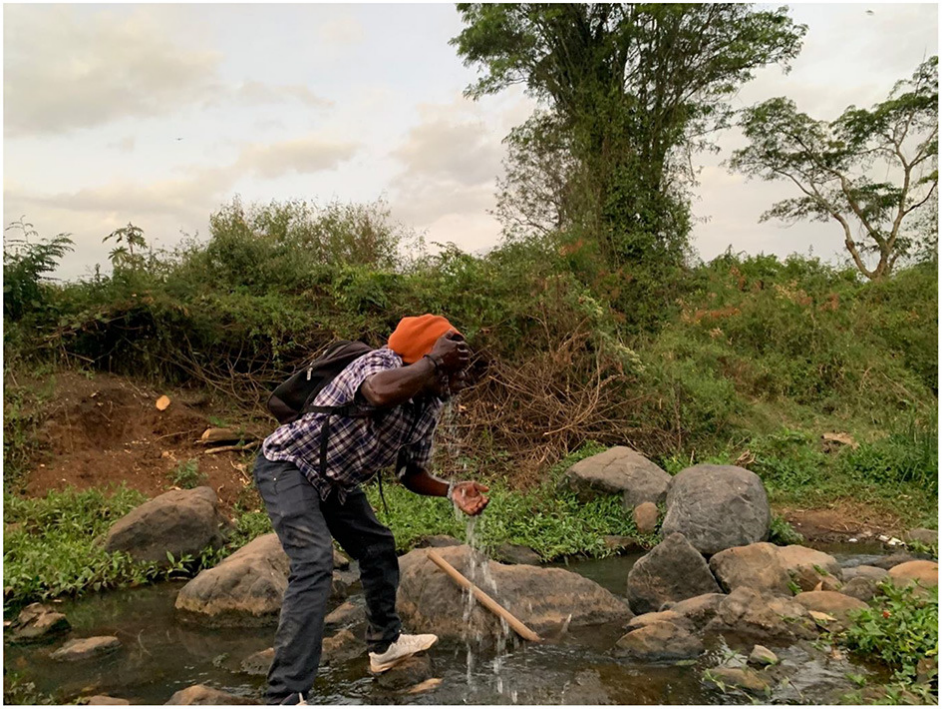
A laborer washing his face after work, Mbagathi River. Authors image, January 2022.

**Figure 2:**
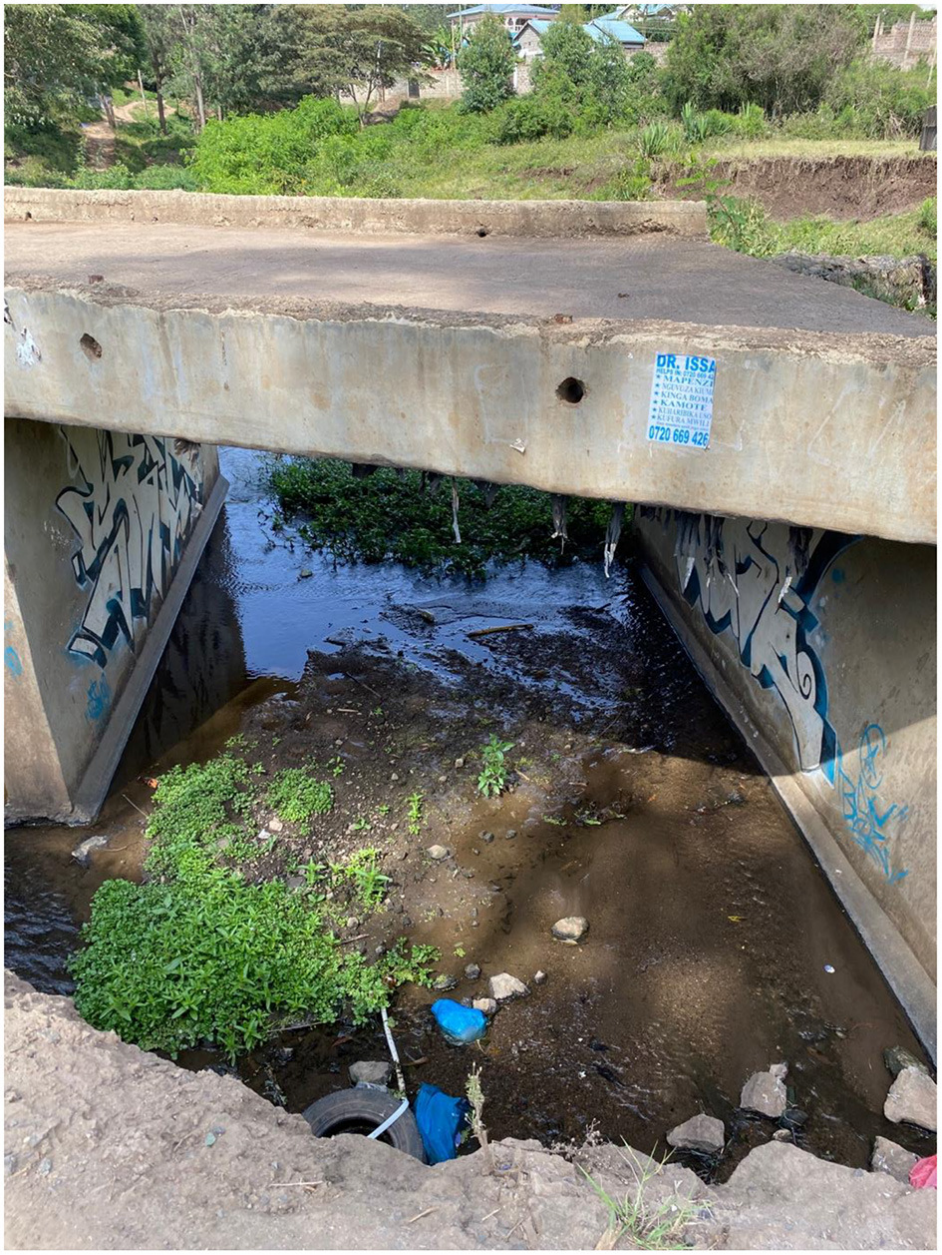
Kandisi Bridge where, in 2018, seven people drowned and 11 missing in a flash flood. The guardrail has still not been replaced. Note at this time of year the water level is very low. Authors image, February 2022.

Riparian spaces are used by farmers, livestock herders, young people socializing, homeless people, as a cut-through by pedestrians, by kids swimming, men bathing after a long work day of laboring on building sites (Figure 1), by women washing clothes, and by people building their homes precariously on the river bank. There is still wildlife: during our fieldwork, a lioness famously wandered through Kandisi from the National Park, and ended up trapped in a small alleyway between two buildings in Kware, and had to be sedated and removed by KWS.^3^ Living on the Kandisi River, I had Sykes Monkeys in my garden, and a huge variety of birdlife including herons and kingfishers. I also witnessed dead fish floating in green and yellow mucus-like scum, vast quantities of “pampers” and other trash blocking river bends and culverts, and shortly before I moved to Rongai, 11 people were drowned on a bridge crossing the river opposite my home when a flash flood caught them and the balustrade they grabbed onto broke off (Figure 2). The river gives and takes away.

My embeddedness in this community and the constant visuality of the rivers in my daily life prompted this study, and the dichotomous nature of the riparian spaces made this an important area for research.

#### 2.1.2. Access to the field

The fieldwork was conducted at the end of 2021 and the early part of 2022. The research team consisted of myself as PI and a resident of Ongata Rongai, Kenyan artist and curator James Muriuki, who documented the process and is also a resident of Ongata Rongai, with assistance from a facilitator from the office of the sub-chief. Having previously worked in Kware, and being a resident in Rongai myself, I had various networks which I was able to draw on for access and permissions, as well as to identify individuals to interview.

#### 2.1.3. Consent and participant information

We created and printed a participant information card with a photograph on one side of the Kandisi Dam, and an explanation of the research project on the other in both Kiswahili and English. As PI on the project, I am bilingual in Kiswahili and English, so all translation was done by me. For interviews, participants were asked to give oral recorded consent since a number of them could not read or write, and asking for written consent would cause unnecessary embarrassment. In every case, participants were given an information card to take away with them. This included a phone number for the project where they could contact us in case of any queries or in cases where they later might decide they did not want their information used. Names were not always written down, to maintain anonymity, and basic demographic information was instead recorded to situate the interviewee, as well as important information about their link to the rivers.

#### 2.1.4. Ethics and approvals

Ethical approval was granted through the ILRI Research Ethics Committee (IREC) approval number ILRI-IREC2021-09 and this was later granted a short extension due to COVID-19 institutional and government restrictions halting fieldwork many times. The project was also approved by the University of Liverpool Research Ethics Committee in May 2021, reference number 9949. In-country permission for the project was granted from NACOSTI, license number NACOSTI/P/21/11430.

#### 2.1.5. Data storage and protection

Data were stored in a password-protected Google Drive and backed up on a 1TB hard drive accessible only by the PI. Only the PI and transcriber had access to these files which included the original audio files, the transcribed files, and the translations. Data are available on reasonable request.

### 2.2. Methods

#### 2.2.1. Archival research

**Figure 3:**
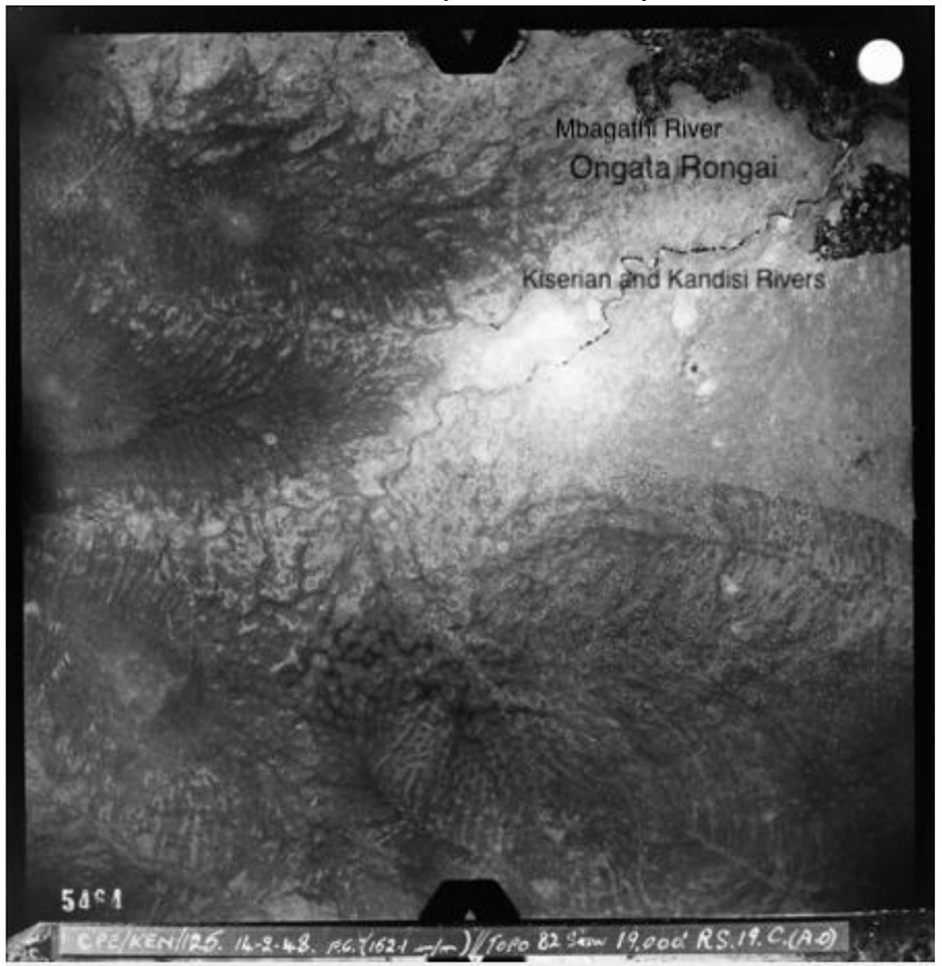
Ongata Rongai in 1948, image courtesy of NCAP.

**Figure 4:**
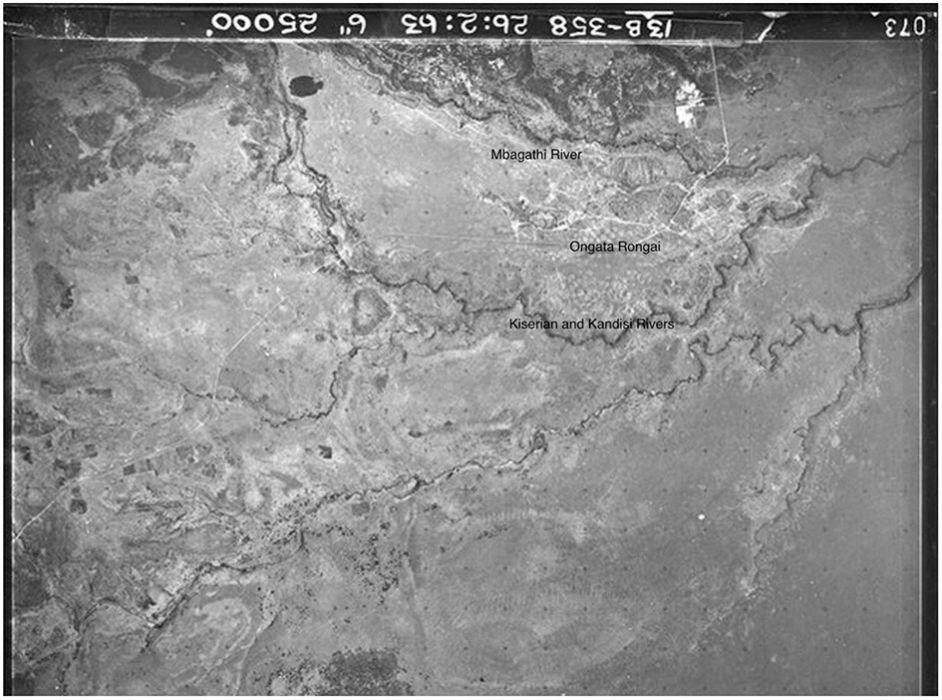
Ongata Rongai in 1963, image courtesy of NCAP.

**Figure 5:**
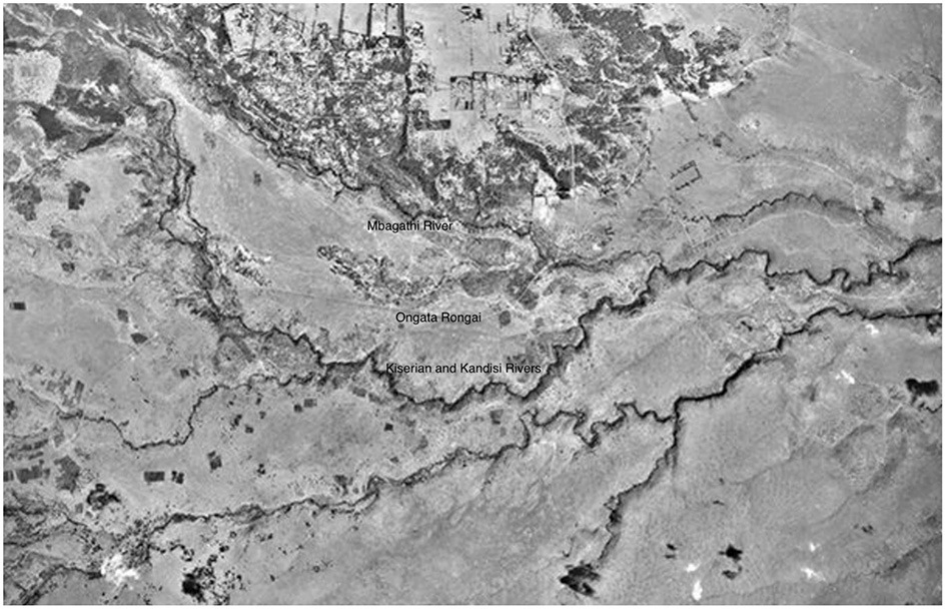
Ongata Rongai in 1969, image courtesy of NCAP.

Before fieldwork, I wanted to undertake archival research to better understand the historical context of the study site. Initially, I wanted to visit the Kenyan National Archives but quickly found that access was an issue, and much of the material we wanted to look at was not digitized and was not organized in a way in which members of the public would be allowed to access. Instead, I contacted the NCAP—National Collection of Aerial Photography in the UK, since they have a colonial-era aerial photographic archive. I was able to search since the majority of their archives are digitized, and they were able to search in other archives which are not yet digitized. These searches produced a number of interesting reference points: aerial images from as far back as 1948 (Figure 3), and other images from 1961, 1963 (Figure 4), and 1969 (Figure 5), which showed the growth of Rongai from no buildings in 1948, to a small cluster of quarries and what might be a group of temporary structures in 1961 and 1963, and the gradual spread of these in 1969, as well as what looks to be more enclosure of land to the south of the rivers, in the Kware^4^ or quarry area, and along what is today called Magadi Road, the main road through Ongata Rongai.

**Figure 6:**
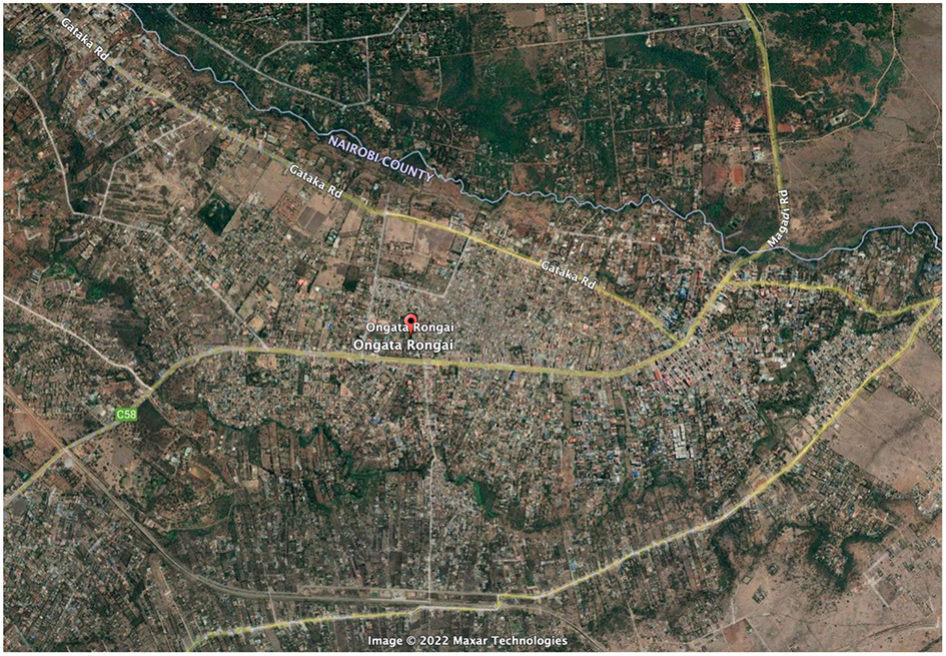
Ongata Rongai in 2022, screenshot taken from Google Maps. Note the boundary between Nairobi and Kajiado Counties is the Mbagathi River, and to the right of the image, the Mbagathi merges with the Kandisi and Kiserian Rivers and continues as the boundary with the Nairobi National Park.

**Figure 7:**
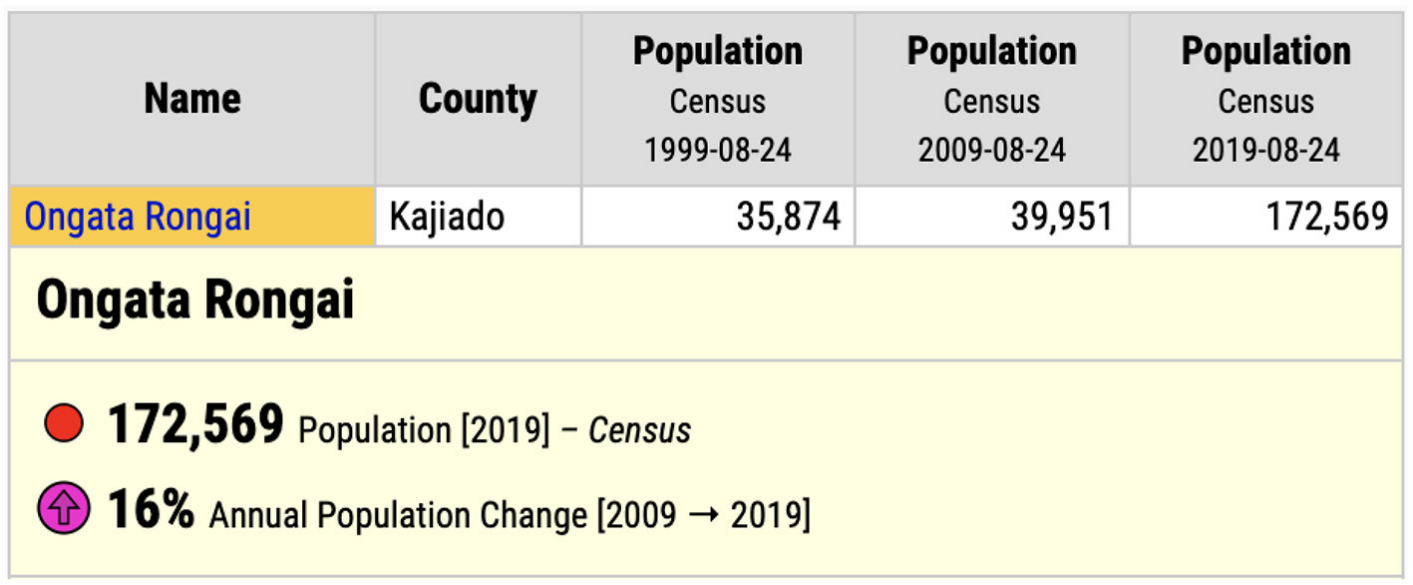
Dramatic growth in the population of Ongata Rongai, 1999–2019. Source: Kenya National Bureau of Statistics.

Finally, we visited BIEA—the British Institute in Eastern Africa, and looked through their library of archival documents, including maps, written reports, and research from the Ministry of Planning and National Development, and the Institute of African Studies at the University of Nairobi. This document allowed us to see that at the time of publication, 1986, Kajiado was very sparsely populated. The report stated that in the “1979 population census, Kajiado District had a total population of 149,005 inhabitants” and that the most densely populated areas were Ngong town, Kajiado town, and Loitoktok. Ongata Rongai is not reported. The mean population of residents in these “urban” settlements was 2,819, but the report predicted a very high rate of population growth, although certainly not to the extent we see today (see Figure 6). For context, as of the 1999 census, the population of Ongata Rongai was 35,874; in 2009, the population of Ongata Rongai was up slightly to 39,951; but in 2019, this had risen dramatically to 172,569 (Figure 7).

**Figure 8:**
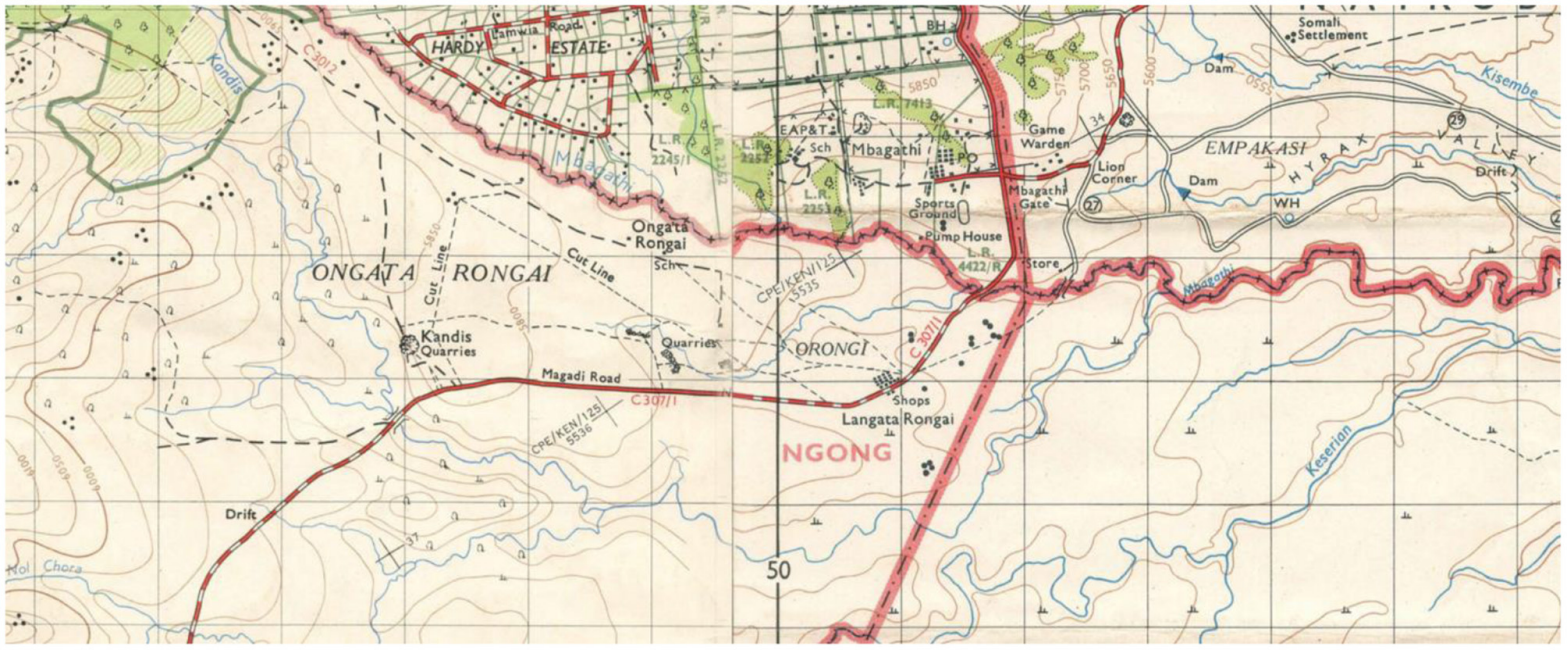
Map of Ongata Rongai published in 1964 by Survey of Kenya, courtesy of the BIEA archives in Nairobi. Note almost no permanent structures, and the same rivers creating the county borders.

The map (see Figure 8 below) courtesy of BIEA allowed for the depth of context, triangulation of participants' narratives in terms of historical context, and reflexivity in interviews as I took a laminated copy with me everywhere to elicit discussion and debate around the changes Rongai inhabitants have witnessed during their lifetimes. Indeed, the purpose of a historically grounded ethnography is both for triangulation and to understand change longitudinally to better relate to participants' experiences and memories and to create more wholistic contextualization, placing the issue of urban rivers within historical and contemporary realities. Archival data were sorted and coded using thematic analysis.

#### 2.2.2. Interviews and sampling

The narrative data came from in-depth interviews with 30 individuals who were mostly selected through purposeful sampling, with assistance from local networks, as individuals who had lived in Ongata Rongai or near the rivers in the study area for more than 10 years, and who had regular interaction with the rivers either through their work or because they live on or very close to them. Occasionally, this was snowball sampling, with one interviewee directing us to another, and other times, this was convenience sampling where, for example, we were at the river making visual art and observations, and found people interested in speaking to us about their personal experiences or connections with the rivers. Interviewees were given a $10 voucher for a local supermarket as compensation for their time and assistance.

Interviews were often conducted on the riverbanks in peoples *shambas*^5^ or compounds, or in a vacant room at the compound of the sub-chief. Occasionally, we went further afield, either downstream to better understand what happens immediately beyond Rongai, or to the homes of those who have since moved out of Rongai but who had been long-time residents there with in-depth knowledge of change over time. Interviews were conducted in English, Kiswahili, or Agikuyu. As the author and PI, I am bilingual in Kiswahili and English but do not speak Agikuyu. Our transcriber was able to translate these instances, and James Muriuki, the visual artist with whom I worked, is a native speaker of Agikuyu and was able to translate during interviews.

Interviewees came from a variety of demographic backgrounds: From wealthy white “Kenyans” to middle-class and working-class Kenyans, Kenyans of mixed ethnic heritage, business owners, farmers, livestock owners, homeless people, young people, old people, people born in Rongai, people who moved to Rongai, people who moved out of Rongai, and people who were married in Rongai. This was deliberate to portray the range of demographics present in contemporary Rongai society and to shed light on the meaning (or lack of) that the rivers hold for peoples' lives.

The interview data, therefore, is not purporting to be a complete or representative picture, but a partial one, albeit representative of these peoples' views at this time. It is a partial lens, but an important one: those with detailed knowledge of the rivers in Rongai, and the changes they have experienced within their lifetimes are important realities to document. They give us an important glimpse into where we have come from, and where we might be heading, in a rapidly urbanizing and changing world, where pressure on water resources and access to safe water is becoming one of the most acute challenges faced by contemporary and future populations.

Interviews were audio recorded, and later translated and transcribed. These transcriptions were then thematically analyzed, a technique commonly used in social science data analysis (23). The thematic analysis was undertaken by first making multiple read-throughs of the transcripts to establish primary themes, and then through closer and more detailed readings, I was able to establish sub-themes. Then, I analyzed the transcripts and sorted data under these themes using a table in Excel, adding the coded sections of transcripts under themed headings to identify the key themes and recurring issues in the transcripts.

#### 2.2.3. Walking, observing

As an anthropologist, I do a lot of walking. Walking is considered part of observing in “typical” anthropological work. However, as part of the Rongai Rivers Project, I wanted to use walking as a more deliberate methodological engagement with this urban landscape. This has been a technique used by social scientists and writers in recent years (24–26) and is an emerging methodology allowing for deliberate explorations, in particular, of liminal spaces. Walking is one of our most basic functions as humans, yet as a methodology, it has been ignored. Walking with an aim or an objective, and walking deliberately, is a neglected but rich source of data. While walking and exploring the riverbanks and riparian spaces, I observed. I observed how people use these spaces, how they traverse them, and how they interact with the river and riverbanks. I observed whether they treated the space with revulsion, reverence, or disregard. Walking was an integral part of data collection in this study. Extensive notes were taken during and after (as context allowed) walking and observations and these notes were thematically analyzed alongside interview transcripts.

#### 2.2.4. Drone flying, plaster casting, and photographing

To get a more complete picture of the riparian spaces around the Mbagathi, Kandisi, and Kiserian Rivers, we worked with a drone pilot. By doing this, we were able to see the rivers in their contemporary context surrounded by high-rise apartment blocks, houses, and in some places, farms. We took plaster casts of the footprints of those who frequent the river including human and livestock footprints. These photographs, plaster casts and drone footage were intended to become part of an exhibition, which COVID-19, funding and time meant did not go ahead. It was, however, useful to get a more in-depth understanding of these spaces. We documented through photography activities taking place along the river and the riparian context and some of these images are present in this article to give the reader a sense of this space. Photographs were used reflexively in interviews to prompt discussion and also were used in this article to illustrate the context of the study.

## 3. Findings

### 3.1. Historical context of the rivers and settlement of Ongata Rongai

Ongata Rongai was until fairly recently a savannah with little or no permanent structures. It was an area where Maasai would graze cattle, bring them to drink water from the rivers, and move around so as not to overgraze one particular area.

One former longtime Rongai resident described his recollection of Rongai “town:”

In those days there wasn't anything really… you'd just come up that hill, and there was like this little group of shops but beyond that there wasn't anything until you reached Kiserian.
(Older male former Rongai resident, Nairobi, January 2022)

And another recalled a similar settlement:

There wasn't a [Rongai] town. There was a little ring of huts. And in the middle, there was the cattle market. And the huts were mainly occupied by prostitutes. So that was Rongai.
(Older female business owner and resident, Rongai, January 2022)

When people began to settle in Rongai, one issue they faced was human–wildlife conflict. The area was heavily populated by wild animals:

You want to know what this area used to be like? You could not pass here alone. We had lions, lions could be heard roaring outside your door. You could hear leopards crying at night. Hyenas, we had every type of animal here. But these days it has changed because people have built everywhere. And the animals, you see this area? It was bush up to Gataka. And when the grass was long it was impossible to pass here alone, because of lions. And you'd ask yourself, where did that lion come from?! But now, you see up at Kandisi, the group of lions has gone up there instead.
(Older female farmer, Rongai, January 2022)
I remember, in those days my mum used to tell me, oh, those animals are destroying my crops, like eland, other antelope. The electricity was not there, these electric cables, so it was put at that time, but the whole area was like, open. And these rivers, you would just cross them, and animals were jumping over them. It was just a wide-open space. So the animals used to come and there was much conflict.
(Older male resident, Rongai, March 2022)

Indeed, this is still an issue for some, and memorable interactions continue to occur now and in the recent past, as well as positive interactions with the river:

Interviewee 1: When we were building we had a lot of [wild animals]. You could find footprints in the morning all over the place. Freely moving around. Like I said, like even where we currently stay, if you follow the river, this is the most shallowest part of the river, where we stay, the river is quite wide when it is full. We used to also do fishing. Like I like fishing, my grandma likes fishing, at that time we used to catch like tilapias, crayfish, carps, catfish, mudfish, then things changed obviously.
Interviewee 2: The river was cleaner, that's why.
(Middle-aged male business owners, Rongai, March 2022)

Although others have some amusing recollections of the rivers in Rongai in the past years:

Anyway, there was somebody, was it an American lady? She had rented [the interviewees Maasai hut] for a bit. And I told her to go, that we could go to the river and swim. And she was delighted. And we took all our clothes off, as we always did. And went swimming but suddenly, out of the cracks in the in the rocks, came a swarm of bees, and they always had bees there. Now, bees don't frighten me. But she went crazy and said I'm allergic now forgotten my *dawa*, my medicine today. And so if they sting me, I die. So I said, right, what do we do? What do you do? And you have to dive down and swim up the river. And we did that, it was very complicated. But the bees lost her. And then—but there we were stark naked. Halfway up the river, our clothes were down there. It was a little bit complicated. Two youngish women, well, not young anymore. Wazungus [white people]!
(Older female business owner and resident, Rongai, January 2022)

Many people recalled the environment as being open plains, with clear and clean water in the rivers:

This Mbagathi River, you used to be able to see the fish, how they were swimming in it. It was so clear, we were drinking the water, washing in it, swimming, watering the gardens. You could see all the stones in the riverbed, it was basically like the water we buy in jerrycans today, that's how clean it was. But now when I think about it, the world is really changing, huh. It used to be as clear as bottled water, imagine, with the fish swimming in it.
(Older male resident, Rongai, March 2022)
…the giraffes, the wildebeests, we could meet in the morning, there were no buildings.
(Older female missionary and resident, Rongai, February 2022)

Some recalled the area being heavily forested:

So in those days you'd find a lot of trees down by the river, many big trees. And people would still graze [livestock] down there. But maybe because it was… you know, the other side was national park, and those game wardens… it meant you couldn't cut trees there.
(Older male resident, Rongai, March 2022)
[in those days] there was nothing here, just forest. No people. No one was living there, allllll the way up to Kileleshwa, there was nothing. No people, just forest. We used to graze our cattle all the way up to Kileleshwa in those days.
(Older male livestock owner, Rongai, January 2022)

But others recall the area as being very dry with little tree cover. This is reflected in the aerial photographs:

In the old days, like when I first came here, you wouldn't see any trees. It was a desert. You know, there just weren't really any trees because it was so dry, it was desert here.
(Older female resident, Rongai, January 2022)

### 3.2. The growth of Ongata Rongai

#### 3.2.1. The historical picture

From the historical aerial images, maps, and interview narratives, it is clear that as a bustling peri-urban settlement, Rongai is relatively new. Indeed, by looking at census data in conjunction with these other data sources, it is clear that the growth of the town is relatively recent, mostly within the last 10 years. However, the census data and narratives do not necessarily concur. In interviews, people believed that the huge growth of Rongai was largely due to the post-election violence in 2007, where people were pushed from their home areas by fighting:

As soon as—you know as, you know Rongai was not very popular and when we moved in we were very new, we didn't have that many neighbors around here. And then people started coming in… [during] post-election violence. That is when Rongai just blew up. Because this was the safest place, there was no issues, infact, even my relatives from Nairobi moved to Rongai to stay with us during that.
(Middle-aged male business owner, Rongai, March 2022)
…but after post-election [violence] that is when things changed, population shot up, prices of land shot up… it was peaceful [here], so you find people had checked out of other places and came here…
(Older male academic and resident, Rongai, February 2022)

Others identified when people began settling in Rongai during the 1950 and 60's, after the Mau Mau uprising:

I think people really came here during The Emergency for the first time.
(Older male resident, Rongai, March 2022)

Others believed the idea of calling one's friends and family was the main contributing factor to rapid population growth, as well as the presence of work and cheap land:

When people began moving here, a plot could be bought for 17,000 shillings [~170USD]. And now you know it is millions. People would call their friends and family and say, hey, come and live here, the plots are so cheap. Seventeen thousand and you'd get a plot… so that's why people just kept coming. They came, many, many, many more. But in those days people mostly came for the quarries [for work].
(Older female resident, Rongai, January 2022)

**Figure 9:**
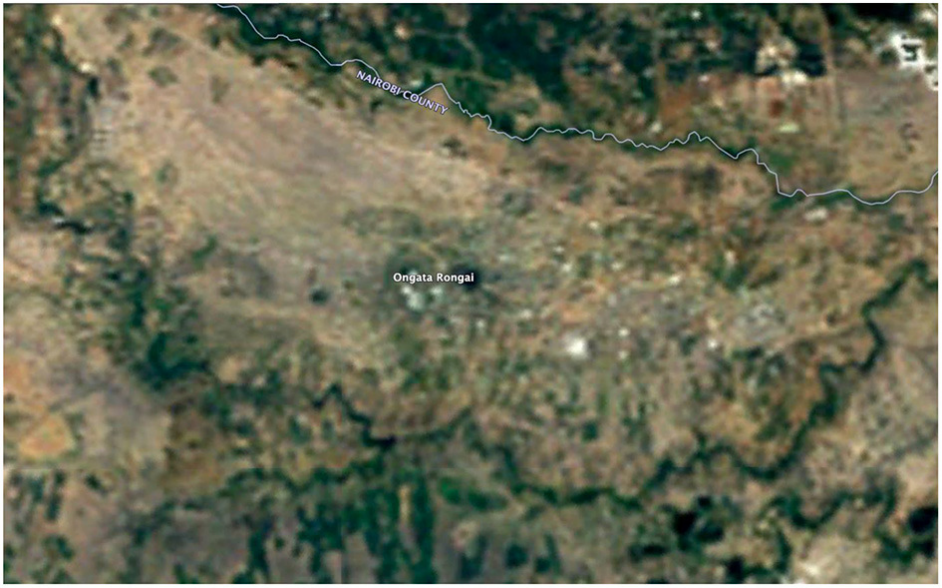
Ongata Rongai 1985, image source Google Earth.

**Figure 10:**
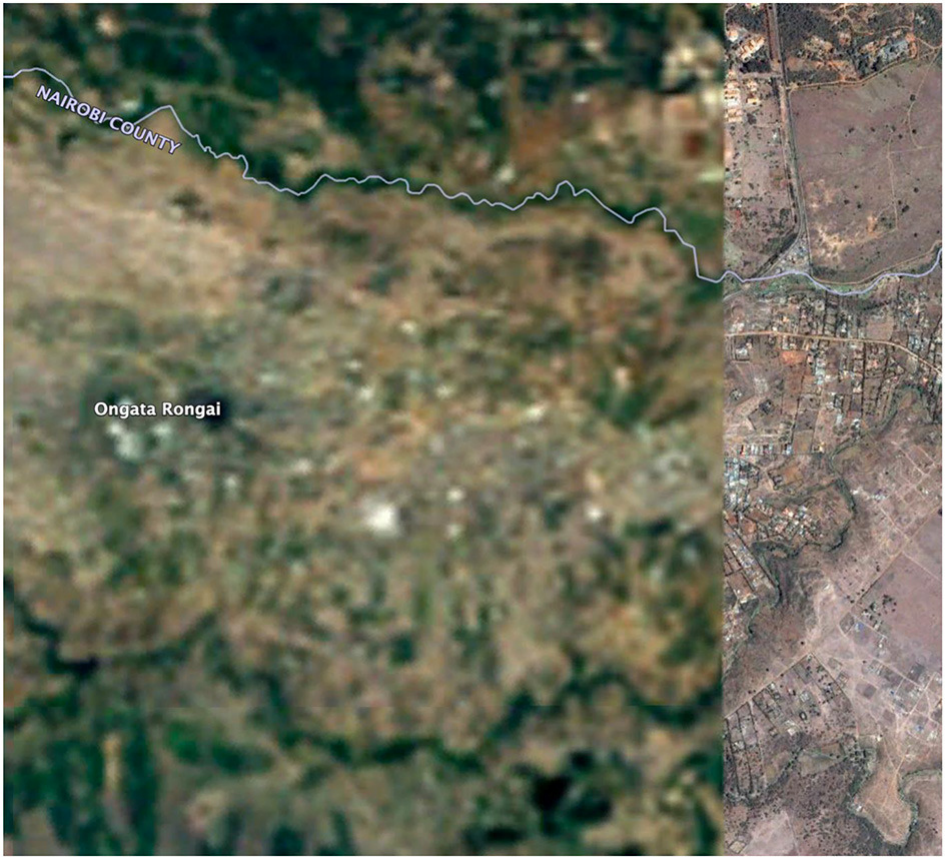
Ongata Rongai in 2002, image source Google Earth.

**Figure 11:**
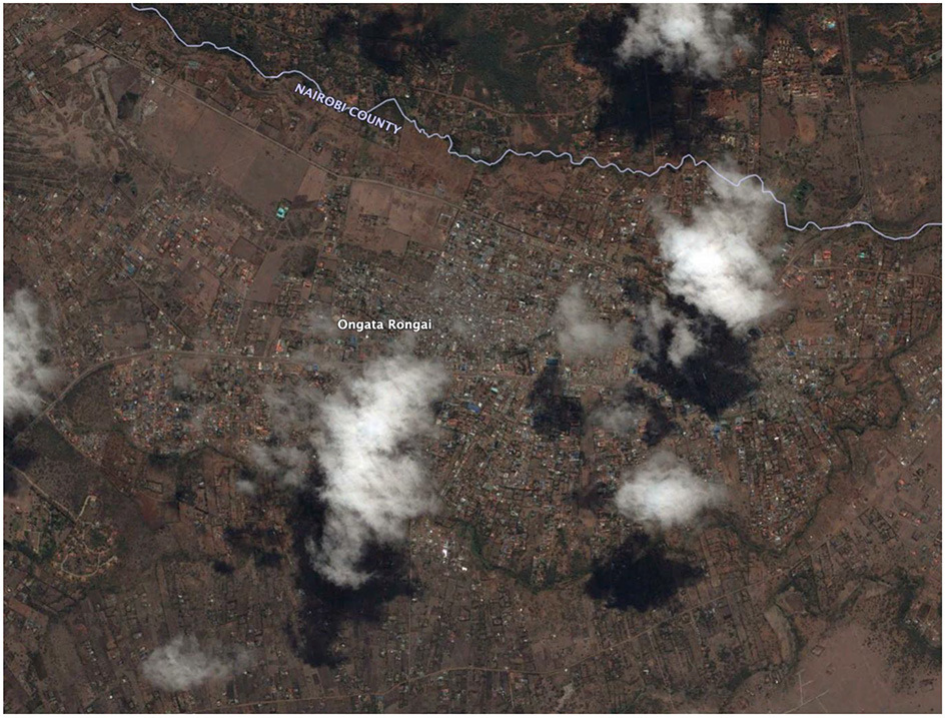
Ongata Rongai in 2008, image source Google Earth.

**Figure 12:**
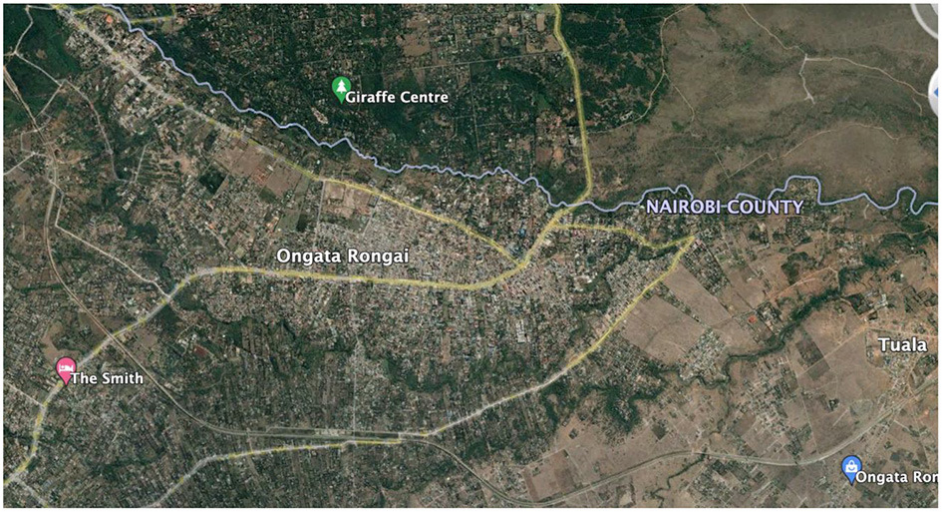
Ongata Rongai in 2021, image source Google Earth.

By examining aerial images from NCAP and Google Earth from 1948 until 2021, we can see this growth occurring, with initial buildings appearing in modern-day Kware, where the quarries are, in the 1960's, and much more development happening in the 90's and early 2000's. Although image quality is very poor, especially for the earlier images, it is possible to get an idea of the development of the town. Although blurry, the image from 1985 (Figure 9) seems to show very little in the way of permanent structures, with development focused still on the Kware area in the center of the image. It is not until the partial image from 2002 (Figure 10) that we begin to see large-scale development with permanent structures and sub-division of land, and from here until the present day (Figures 11, 12), the rate of development is rapid.

This recent and rapid development was supported by narratives and by census data, and as I will go on to discuss, services and, in particular, waste and water management have not kept pace with this level of population growth.

#### 3.2.2. Rongai and the rivers today

As demonstrated by the census data and aerial images, Rongai is now a densely populated urban area, no longer peripheral to but a part of Nairobi Metropolitan. This was clearly reflected in narratives, where people talked about how the “old” Rongai was very much in the past. Rapid development without services (mains sewage, solid waste disposal, piped safe water, or any sewage treatment plant, as well as roads, electricity, drainage, or healthcare services) keeping pace has contributed to a litany of issues for the health of humans, animals, and the environment:

For the environment… it really started to change when these very big buildings [apartments] began being built. And then people began calling Rongai “the sewage place”… and so that's a big problem… people will open the sewer into the river at night… and this problem has come because the population here is so high and there is no plan for sewage… and now if you go and see, that water [in the river] is sewage only.
(Older male resident, Rongai, March 2022)

Indeed, the way in which riparian spaces and river water are used has been forced to adapt. People are very aware of water safety and are wary of using river water for any purpose:

The problem with this river is, it is filthy. You see, if you use it to water your vegetables in your garden, even you are eating that filth. If you harvest these vegetables right now, you go and cook them, you are eating that filth. And it's not even just us, it's the animals too who have to drink that water. So those who are pouring the raw sewage into the river, can they just think about it and stop?
(Older female farmer, Rongai, January 2022)

Yet, some people are forced to use the river water, for farming, laundry, bathing, watering livestock, and other purposes, often out of necessity or convenience.

These days if you wash in the river you find your skin dries up… as if you have an illness… and if you keep washing there, you'll get typhoid.
(Older female farmer, Rongai, January 2022)
Interviewer: ok so these days what do people actually use the water for?
Interviewee: laundry, only laundry. And maybe herdsmen take their livestock there but it's no good for them, some refuse and the smell of the water is terrible.
(Older male resident, Rongai, February 2022)

We witnessed women doing laundry, adults bathing, children swimming, and livestock drinking at the river, but narrative interviews clearly stated that people are aware of the potential risks of doing so. Uses of the river have changed, but some people are forced to depend on the river and riparian environment for their livelihoods.

### 3.3. “Kenya is just heaven and hell side by side isn't it, in many ways?” the challenges of the Mbagathi, Kandisi, and Kiserian rivers

#### 3.3.1. Sewage and poisoned water

The main problem of the rivers today, cited by everyone we spoke to, was that of sewage. Untreated sewage is pumped into or flows into the rivers daily, primarily from low-cost and poorly constructed apartment buildings belonging to wealthy businesspeople. Interviewees narrated how apartment blocks and businesses release chemicals and organic waste into the rivers regularly:

…3 years ago, suddenly the whole river was full of floating dead fish. Because so many of the businesses up the road, they had released, *chemicalized* sewage at the same time, the whole river, they poisoned the river completely… and we are talking thousands of fish floating upside down… people with flats, you know, those high rises, just across here.
(Older female resident, Rongai, April 2022)

Yet, institutions and wealthy homeowners have also been known to release sewage into the river:

Even that university [redacted] they are pouring raw sewage into the river, directly! They were told to stop, but before that they were pouring it straight into this river.
(Older female farmer, Rongai, January 2022)
Up there is [senior government official/redacted]'s house. Even he was pouring sewage into the river. But he was stopped because of the river. But even there is [another senior government official/redacted] and he was doing the same thing. They don't care.
(Older female farmer, Rongai, January 2022)

**Figure 13:**
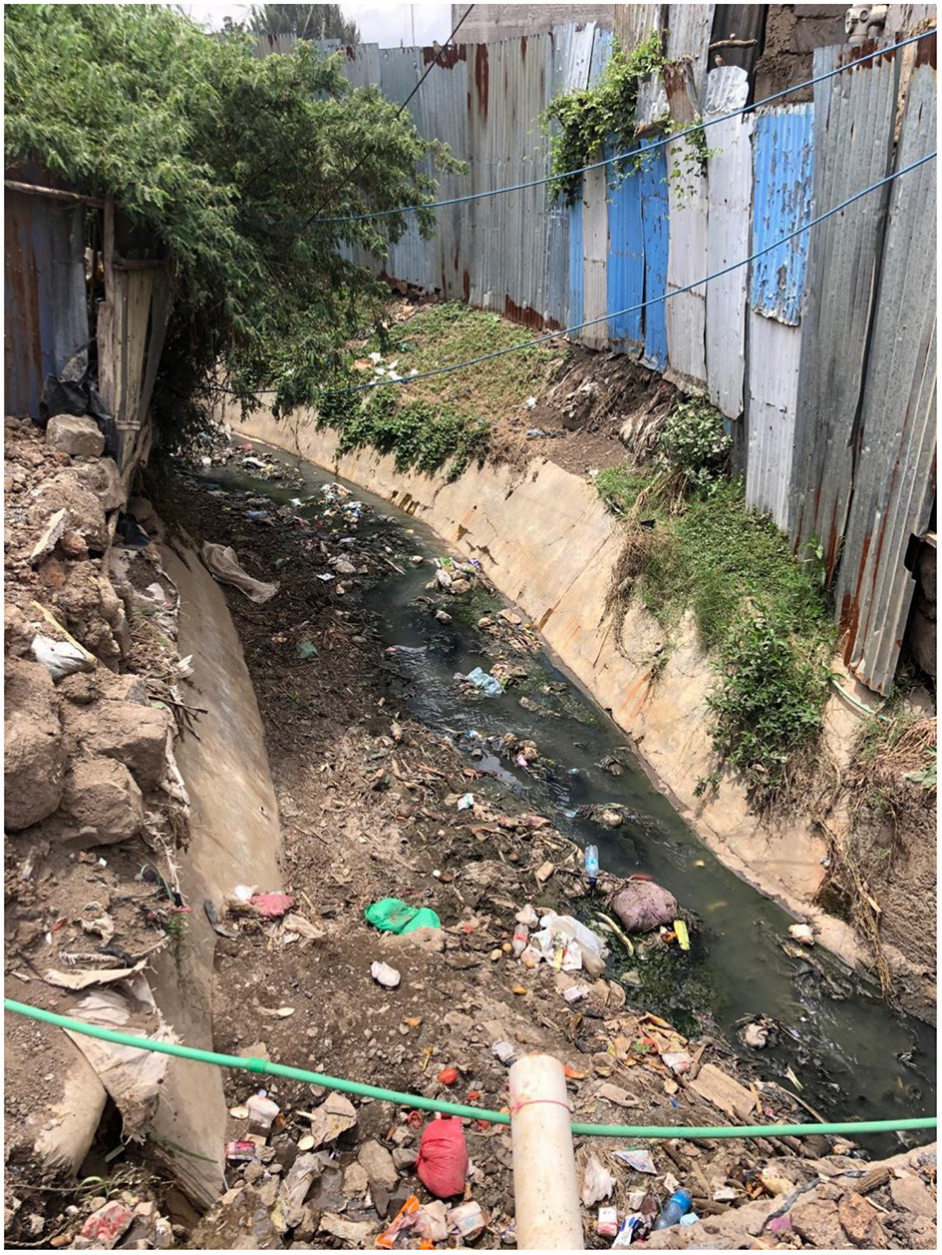
Trash and sewage flow into a culvert in Kware which meets the Mbagathi River close by. Authors image, January 2022.

The anecdotal presence of high levels of sewage (although sewage and solid waste were observed—Figure 13—and smelt in almost all locations) and chemical waste in the rivers has affected businesses as well as individuals:

Pollution *is* the river, man. It's really messed up. Especially for us, even for the restaurant, because what people are doing, they are building these high rise apartments and stuff, and the sewer system is directed right into the river. So when it rains, the evening hours, that's the time, *wanafungua* [they open] all the sewer systems so it goes through into the river and for us guys because we are next to the river, we get a strong smell of sewer, you know like, it's like you can literally get ill. If you sit at the table, we couldn't put clients on the terrace because it was so strong, the smell. So that was a big issue.
(Middle-aged male business owner, Rongai, March 2022)

Sewage has severely affected the health of the rivers and has a knock-on effect on livelihoods and human health upstream and downstream:

The fish are affected by this sewage. They get confused and swim on their sides… Like now you'll find the tilapia are very small, unusually small. Because there are chemicals in the river water… it kills many of the fish. And people were just collecting them [off the surface of the water]. Some they say were spoiled, others were eating them. So that sewage really affects the water.
(Older male fisher, Mbagathi River, January 2022)

For livestock owners, the water quality in the Mbagathi and the Kiserian Rivers negatively affects their animals:

Interviewer: in those days did you ever find your livestock would get sick from drinking the river water?
Interviewee: no, never, because that water was flowing, maybe from standing water.
Interviewer: and these days, is it different?
Interviewee: yeah, it's different because these days people are dumping sewage there. The water is very little and the sewage is a lot. So now yes, I have seen a lot of change.
(Older male livestock owner, Rongai, February 2022)

The presence of sewage also has a negative effect on wildlife:

You know there are hippos living here, and when there are chemicals in the river the hippos look different. Their faces change. Their skin changes color.
(Older male fisher, Mbagathi River, February 2022)
…when the game drops dead, and when they do autopsies, they are saying, oh god, this is – the water is not good. Or you know, before the wildlife have the dams to drink from. And they are clever, you know, they are sentient animals but… they have nowhere else, you know, a dry year like this year…
(Older male resident, Rongai, February 2022)

There is a significant reported problem of trash and solid waste in the river too, as people see it as a place to dispose of, and carry away, unwanted items at the end of their useful lives:

What is really bothering us is the dirtiness of the river these days, you know, and we have a man… who is almost daily, removing … tires, socks, shoes, clothes, plastic bags… Flying toilets, plenty and this is a concern.
(Older female missionary, Rongai, February 2022)

#### 3.3.2. Structural negligence

The much-lamented issue of a lack of enforcement of existing environmental regulations and corrupt government bodies also featured strongly in interview narratives:

We got guys from NEMA [National Environmental Management Authority] to come and shut them down and everything, but nothing. We have been, at least, we have had NEMA here like four times, but they just come to harass us instead of getting the right people.
(Middle-aged male business owner, Rongai, March 2022)

Frustration with an inept regulatory system was a common theme, and interviewees largely felt hopeless as individuals without a functioning institutional-level framework for environmental protection:

I think what is going to happen [to Rongai] even these issues will become even worse. I can't see that we will ever see the water getting cleaner. Because with this issue of sewage, the way that Rongai has no system of sewage, no planned system. And all that filth just goes directly into the river… it won't be resolved now, not unless people are utterly committed. Like this sewage issue… if it was properly planned and it became law that it is illegal to dump -sewage, people get prosecuted, they become afraid, that is when things will change.
(Older male resident, Rongai, March 2022)

Some interviewees said that local and institutional attitudes to the environment contributed in a systemic way to environmental degradation and destruction:

No one is looking after the river because even the big people don't bother… and there is no plan, no proper plan. I can't tell you that there is a plan [to clean up the river] because there isn't one, and no one cares.
(Older female farmer, Rongai, January 2022)

Others saw this as part of a globally moribund attitude to the environment:

How come there has been 26 of them [COP conferences]? I mean these fucking talking people are useless aren't they?
(Older male resident, Rongai, February 2022)

### 3.4. Climate and environment

#### 3.4.1. Climate change

There was significant recognition of climate change as a contributing factor to environmental destruction in Ongata Rongai. It was noted that water levels and the quantity of rain were factors contributing to change:

This river, slowly it is being finished… it used to be a permanent river but these days you will start to find it is just a seasonal thing. And in many places now it is just full of sediment.
(Older male fisher, Mbagathi River, November 2021)
You know usually this month of November, usually you will get rain in November. But this year even the signs of rain are not there. But this time last year the river was full, it was totally full, so full that it burst this wall and the water was inside the compound.
(Older male fisher, Mbagathi River, November 2021)

Greater instances of human–wildlife conflict were also blamed on climate-related changes:

These days we find many monkeys, colobus monkeys, come and steal crops. Baboons too. They are so many, and such a nuisance. In the old days they never came. Even the small ones, the vervet monkeys. They came because of hunger. They were pushed from far away. They were forced to come here because they were hungry.
(Older male resident, Rongai, March 2022)

Overgrazing in conjunction with drought was blamed for degradation:

The soils are ruined from overgrazing, even the trees now need to be watered. The whole place needs to be left alone, let the trees drop leaves, let the grass grow, then it will be like compost… the soil will regenerate.
(Older male resident, Rongai, March 2022)

In conjunction with climate change, there was an awareness of the overuse of land for farming, as well as the change in rainfall patterns:

Maybe the reason why the soil has no fertility now is because of over-farming it. Even your clothes, if you wear them every day they will wear out, won't they?
(Older female farmer, Rongai, January 2022)
These rains we are getting, you know it rains for maybe 1 day. That is not rain for farmers.
(Older female farmer, Rongai, January 2022)

There was a recognition of the environment slowly worsening, as well as an acknowledgment that this was due in large part to human intervention, but that people have agency and can make positive changes to the environment despite structural-level economic challenges:

Poverty is a motivation for this environmental destruction. If you hear over there people are clearing trees and making charcoal, cutting firewood, selling charcoal, selling firewood, also poverty contributes. But also peoples' ignorance. So actually it's not really poverty, because if people understood the importance of these trees, they would look for another way to find an income.
(Older male resident, Rongai, March 2022)

For our interviewees, the outlook for a climate-changed future was a rather dystopian one:

What will this place be like in 20 years? It will be a desert. A desert.
(Older female farmer, Rongai, January 2022)
We are finished. We will be like the dinosaurs. We will be extinct.
(Older male resident, Rongai, March 2022)

#### 3.4.2. Pressure on water resources

Water is becoming scarcer in Ongata Rongai, as we observed at the rivers and as narrated by interviewees. Often the rivers were little more than a trickle of water, a seasonal stream, rather than the abundant rivers people described from the past. Yet, at other times, the rivers became dangerous, washing away farms and people.

These days the way people are coming to farm, the river is refusing to flow. Even those days that dam used to have plenty of water in it but now you find there is very little. People are not getting water. But long ago there was plenty.
(Older male livestock owner, Rongai, January 2022)

There is an understanding that much of the pressure on water resources is due to human activity:

And now people started farming with machines, irrigation with the generators. So you find this water in the river is finished! It is chaos down there, people fighting over water. And the water, there isn't any left.
(Older male business owner, Rongai, January 2022)

**Figure 14:**
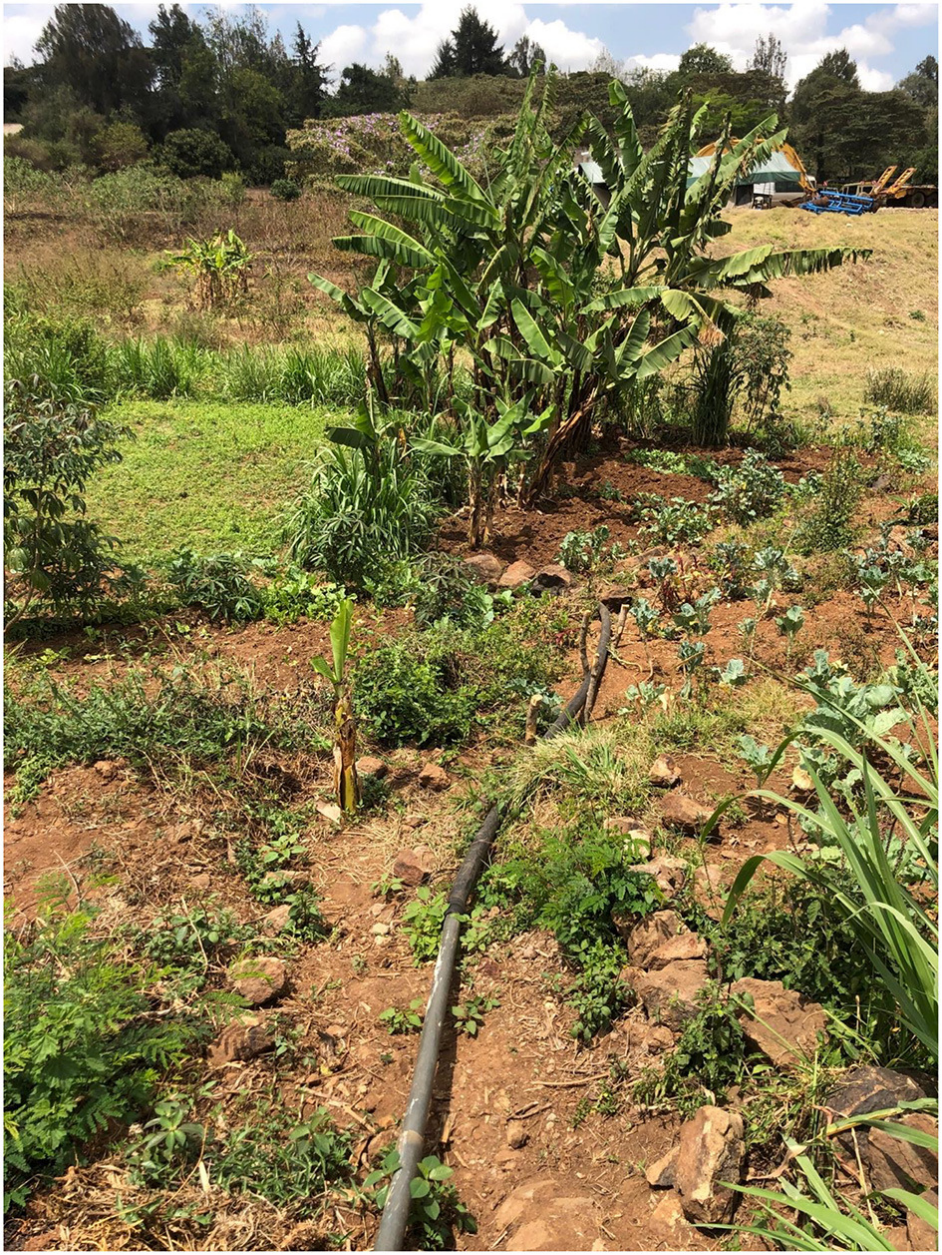
A typical farm on the banks of the Mbagathi River, in Rongai. Note the irrigation pipe in the foreground which extracts water using a generator-powered pump from the river to irrigate crops. Authors image, January 2022.

Interviewees explained that it is not only small- and medium-scale farmers extracting water (Figure 14) leading to scarcity, but large-scale farms too:

These changes really began in the 90's, and I heard much of the changes in the river also happened because of flower farms, like they were diverting water… those farms in Karen.
(Older male resident, Rongai, March 2022)

There was again a sense among interviewees that regulatory systems in the past were more effective than those today:

Even my father used to apply for that permit… we used to use our hands to water the crops but then he bought a pump and he was told, this permit is a requirement and there was an extension worker to explain what you are supposed to do. And the government wanted to control water because if people used it too much then people and even animals would lack water.
(Older male resident, Rongai, March 2022)

Again, there was a sense of frustration with government-level systems being inadequate to oversee water use which interviewees said led to increased pressure on what little water resources remained usable:

As far as I know, if you live along the river, you are allowed to take water out of it, unless there is a subsequent agreement, most parts of Kenya they have water users associations, where it is metered, it is strict, but it doesn't really work because it is Kenya.
(Older male resident, Rongai, February 2022)

### 3.5. Culture and meaning of the rivers

#### 3.5.1. Cultural meanings and change

In interviews with older people, a very interesting idea was discussed: that part of the reason for the environmental destruction which we see today is due to the mixing of many different tribes:

This is the problem. These days, how many tribes are there here? Many. In the old days, how many tribes were here? Two. Two. Which tribe will listen to the tradition of another tribe? None! The problem is, none of those people understand each other.
(Older male livestock owner, Rongai, January 2022)

Generally, people acknowledged that the ways in which riparian spaces were interacted with in the past are now considered history:

Those elders long ago, they would take a sheep to a fig tree [*mugumo*], they would make their prayers there. Then when they had finished, they would leave and the rains would come.
(Older female farmer, Rongai, January 2022)

We were often told that the fig trees on the riverbanks were of great importance to the people of Rongai:

The fig trees by the river, those were the churches of the elders long ago.
(Older female farmer, Rongai, January 2022)

Some interviewees understood that the loss of cultural significance of these places is due to colonial missionary intervention, but that in past years, these riparian spaces were of great importance for ritual and prayer:

In the old days, my grandfather used to tell me, the rivers would dry up. So then the elders from this side and from that side would come and meet and the river. They would bring a goat, and do some rituals with the goat and then the rains would come and the river would run full again. But these days no one does this. Now we are all in church.
(Older female farmer, Rongai, January 2022)
Instead of those traditions, now there is church.
(Older male resident, Rongai, January 2022)

There was a feeling among interviewees that respect for the environment had been lost through the changing of religion and the move away from “old ways:”

If you cut that tree you would be cursed. If you were cursed you would have to come back to the tree, to pray for forgiveness, and bring a cow, you would have to slaughter that cow, and beg not to be killed. But these days people don't care about those things.
(Older male livestock owner, Rongai, January 2022)

Another aspect of cultural change which our interviewees discussed was a generational loss of meaning:

[our children] they do not have time to ask things about the environment. They are digital. This generation are just on their phones throughout, that is their only activity. They don't care [about the environment]. You see people these days, this digital generation, even if they are crossing the road they don't know if there is a car. They are on their phones, and with those headphones in their ears. So now you are wondering, is their brain even functioning properly? It's not. It's problematic. If they get hit by a bike or a car it's because the brain is concentrating here [on the phone].
(Older male business owner, Rongai, January 2022)

And a lack of interest in a shared history and custodianship of the environment:

Interviewer: so the knowledge we have been discussing about the environment and the history of Rongai, young people aren't interested?
Mzee: if they had been here they would have left ages ago because you would have been boring them!
(Older male business owner, Rongai, January 2022)

Again, we heard the same idea of the mixing of tribes and cultures leading to a dilution of morals and loss of meaning:

In the old days, kids could not just go near elders. It was not allowed. But now it's not that way. Now kids speak to elders however they like. It's because there are so many people now, so many tribes, so many different cultures and ideas. It's become now that people are just mixed up and they take on the behavior of others… in this town there is no tradition, there is just confusion, people just do as they like.
(Older male livestock owner, Rongai, January 2022)

#### 3.5.2. Indigenous medicines and plants

I have previously written about indigenous medicines in Kenya and specifically in Ongata Rongai [cite]. For this study, I specifically asked about any medicinal plants growing by the river because I had previously been informed that with the loss of riparian and green spaces, people living in urban areas had increasingly less access to herbal medicines than in previous years.

Interviewees confirmed that there were and are medicinal plants growing in and around the riparian spaces in Rongai:

It was like this, that there were trees which grew near the river there and we would use them to treat various illnesses…
(Older male livestock owner, Rongai, January 2022)

But access to these herbal medicines is not as egalitarian as it once was:

In the old days there were trees and shrubs we used as medicines. Many of them. Like, when you start to get old, there was a tree to help sore knees, back pain… it is called *muteta*. Then another which did a similar thing which we call *Kererwa*. You make soup with it and drink. It's really good for joints. But now it is no longer here. If you cross the river [to Karen] you might find it still. People used to see the importance of those, as in you could not cut those because they were medicinal… but now people prefer the white peoples' medicines. And naughty people will sell these local ones, they cut them, put them in a bag and sell them! In the old days they could not be sold. There were so many, and everyone had an understanding of medicinal plants. So no one would sell. Capitalism has ruined everything. But actually those modern medicines, most come from plants anyway.
(Older male livestock owner, Rongai, January 2022)

#### 3.5.3. Security

In addition to the dangers posed by interactions with wildlife, there were instances where people had heard about or personally experienced attacks from other people. There was a sense of fear about the riverbanks, perpetuated by actual occurrences:

There is a time I remember a while back when young men would go into those farms by the river, into the forest there, and would catch women and rape them… people began to be scared to go down there, it was totally terrifying. It was so bad.
(Older female resident, Rongai, January 2022)

Much of this fear seemed to be anecdotal:

…it is not a safe area also ‘coz you find all the youngsters around there and it is easy for people to, like, steal…
(Middle-aged male business owner, Rongai, March 2022)

Although very frightening episodes occurred for others living directly on the river:

So they started chopping them, the door, with an ax and every time they hit the ax in, I put a hockey stick behind the ax so they cannot pull it back… So while they are chopping the door, I find the safe key, and I take out the bullets which were bought in '82, and load a clip and pass it to him… So he basically fired six rounds into the kitchen and suddenly there was total pin-drop silence. There was nothing in sight. People were running outside helter-skelter and by then I have already gotten another clip ready, pass it to him, he loads it… the good thing is that one of them died on the spot here, and another one died by the quarry and two died in hospital…
(Older female resident, Rongai, April 2022)

**Figure 15:**
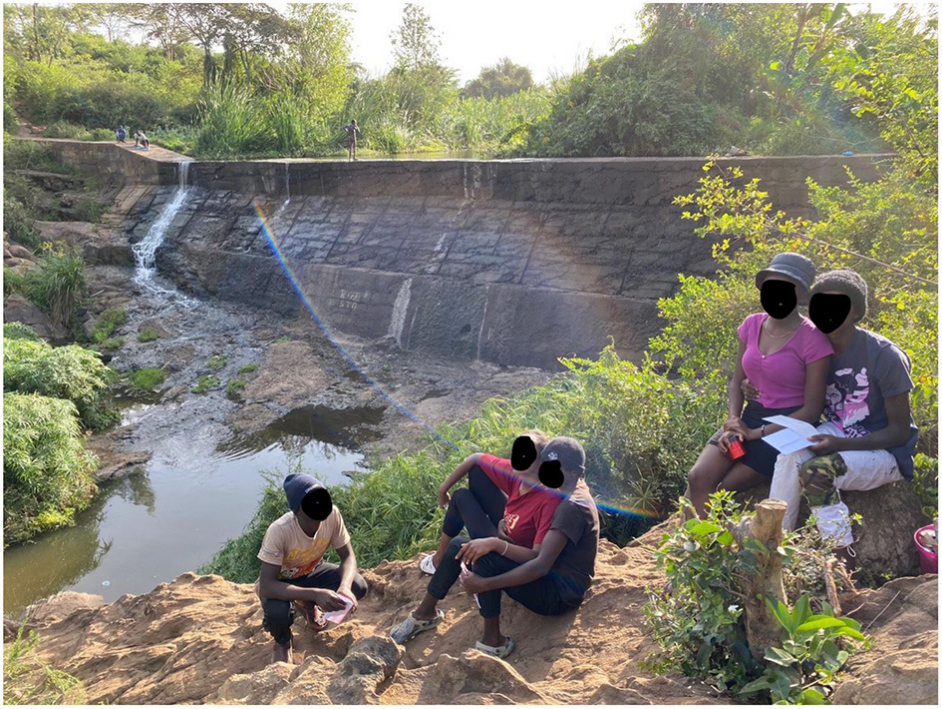
Young Rongai residents “chill” by the Kandisi Dam, and behind them a homeless man conducts his ablutions on the dam wall. Authors image, February 2022.

We observed many young people enjoying these marginal, liminal spaces of the riverbanks (Figure 15). One group was making TikTok videos and hanging out with their girlfriends and boyfriends, and others (a younger group of boys) were swimming, jumping into the water, and using 5L water bottles tightly sealed as float-aides.

Yeah, so we come here because, this place is really beautiful, right? Yeah. We like nature, don't we? So we come here and chill and make TikToks. So we walk around the neighborhood, try and find somewhere to make TikToks, and this place, the waterfalls, it's cute… he does the [video] shooting, then she uploads, then we choreograph… we don't have a camera so… we just use the phones… because we already found a cute place, there's no need to go anywhere else, and here's cute, right? So yeah, we post on Instagram too… Facebook… we get more likes when we post stuff from here, so we just prefer to make TikToks here now… there's crocodiles here but they're ok… we don't get anyone telling us not to film here, or not to do this or that, so it's cool here… and some people come to smoke weed [laughter].
(Teenagers, Rongai, February 2022)

We spoke to a homeless man who said that, for him, the rivers were an important place:

I come here to bathe, but usually I sleep just under a shop roof if I can find one… I have probably been coming here for 3 years to bathe, even if I find a place to sleep, I still come here to wash… I wash my clothes, and even this river water, I drink it… this water is clean, it is clean… I have never been sick from using it, never, and I really depend on it, you know?… and if I find anyone *dirtifying* this water, I get very angry, I feel very bad about it… and you know, there was a time when I don't know where it came from, upstream, but the water was poisoned by sewage and it turned black… I felt such pain, to see the river which provides for me being poisoned… but God made this river… therefore if God made it then it will continue forever.
(Middle-aged homeless man, Rongai, February 2022)

## 4. Discussion

### 4.1. A rapidly urbanizing space

From our fieldwork, archival explorations, and census data, it is clear that Ongata Rongai has experienced dramatic and rapid change over the past decades, especially in the last 15–20 years.

In the past, people would collect medicinal plants from the riverbanks, and indigenous religious ceremonies were conducted there. These rivers and their immediate surroundings were of great significance to people's health and spiritual wellbeing. This is no longer the case. Christianity erased indigenous beliefs, while concurrently erasing traditionally held proscriptions and taboos which preserved the environment.

The riparian spaces were, in the past, dangerous for different reasons: wild animals abounded such as hyenas and lions, and human–wildlife conflict was common. These days, the most common threat is from nuisance monkeys and baboons destroying crops, but there are still occasional encounters with lions or hyenas, especially in riparian spaces, giving rise to a sense of wariness.

This rate of population growth, while understandable as people from rural areas attempt to seek employment and provide for their families in urban spaces where there is a perception of financial gain, is surely unsustainable. The current rate of population growth of 16% annually seems likely to rise. It is unclear whether a tipping point has already been reached but it is clear that if current conditions continue, the Ongata Rongai environment will soon reach its carrying capacity. Narrative, census, and archival data all show that the change in population in Rongai has been rapid. The deleterious effects of rapid population growth and urbanization on health have been well-documented (27, 28), especially where systems do not keep pace with this growth.

Basic systems and services such as water, sanitation, electricity, and sewage treatment have failed to keep pace with this level of development, and residents are bearing the brunt of this. Official individuals and agencies seem incapable of supporting the community in any meaningful way and “talking people” are, as several interviewees noted, “useless.” There is a feeling that people are thoroughly fed up with words and now action is required to provide basic services to the people of Ongata Rongai to maintain health and wellbeing. Many interviewees are aware of the risks posed by the lack of access to basic amenities such as clean, safe water, and sewage and solid waste disposal. Not only does this affect humans but this is a quintessentially One Health problem: humans, animals, and the environment are intrinsically linked, yet the data show that the environmental degradation being experienced in Rongai is leading to poor health outcomes for both humans and animals. These issues, when combined with the pressure of demographic growth on resources, heightened the risks to human, animal, and environmental health.

### 4.2. Users and uses of the rivers

Historically, the Mbagathi and Kiserian/Kandisi Rivers were places of significance for humans and animals. People worshiped at the *mugumo* trees, drank the water, farmed, fished, washed their bodies and their clothing, brought their livestock to drink and eat, collected building materials, and gathered healing herbs. The sacred significance of riparian environments and especially *mugumo* trees is well-documented (29, 30).

Today this has changed. Pressure on water resources caused by rapid population growth has led to visible pollution and degradation of the environment. Fish die, livestock gets sick from drinking the water, and some even refuse to drink it. People still (rarely) use the water to wash their bodies, but most understand the risks of drinking or having contact with the water from these rivers (20).

People's capacity to grow their own food, catch fish, provide water and fodder for their livestock, and perform basic hygiene functions such as washing their bodies and laundering clothing is all impacted by the current state of the Mbagathi and Kandisi/Kiserian Rivers. This is a problem both upstream and downstream, and indeed those living downstream were further impacted by industrial waste from the Embakasi area of Nairobi flowing into the Mbagathi River. All along the rivers, wildlife graze and drink, and their health and wellbeing are dramatically impacted by the lack of access to clean water and trash-free grazing. Interviewees described how livestock refuse to drink the water, or get ill from doing so, and crops shrivel and die. This forces people to look for other sources of water, which is expensive and, for many, impossible. Crops watered with contaminated water can cause slow poisoning by heavy metals (31, 32) and knock-on contamination of soils through leaching (33, 34). This causes the slow poisoning of those who eat the crops, including the destruction of the metabolism, damage to DNA, and associated cancers (35).

**Figure 16:**
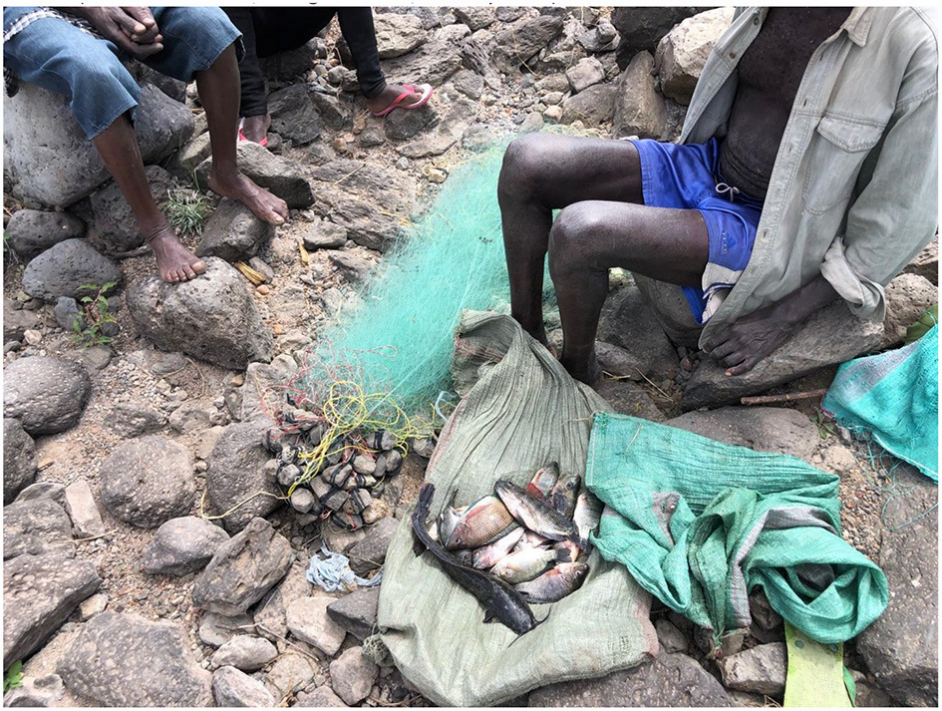
Luo fishermen display their catch Mbagathi/Athi Rivers confluence. Authors image, January 2022.

Many farmers have lost their livelihoods: they may still farm on the riverbanks, but their crops suffer from the poor water quality and yields are low. Fishers (see Figure 16) report reduced catches, “confused” fish, and sometimes skimming the dead fish off the surface of the water, and the dangers to fish and subsequently to the humans or animals who eat them have been discussed (36), and indeed the risks of transfer of antibiotic resistance (37). Some people eat floating dead fish, as interviewees reported, yet others understand the dangers of doing so.

Sometimes you still see women washing clothes, bedding, pots, and plates in the river. Many people understand the risks of doing so (20) but perhaps have little choice since the cost of purchasing water is beyond their reach. Medicinal plants are fewer these days, and urban dwellers struggle to access anything other than allopathic medicines (38).

For many, the rivers are merely a cut-through, a quicker way for pedestrians to reach their destination. Indeed, their interest in the rivers is minimal, and their interaction with the riparian environment is fleeting. Wild animals still occasionally roam the riverbanks, adding to the sense of unease, and encouraging only fleeting interactions with these spaces.

The rivers for some are a conduit for the removal of trash, dirt, excrement, and other unwanted things (39, 40). This transposition of meaning from sacred to profane is a result of the loss of cultural significance: interviewees cited the proliferation of Christianity—“the religion of the colonizers”—as responsible for this. Since the rivers hold no meaning, is it any surprise that they became a dumping ground for the unwanted? Crossing the boundaries of clean and unclean, the rivers mark a liminal, contested space, one where “traditional” livelihood activities persist, despite challenging conditions, and where working-class Kenyans confront the waste of the wealthy.

For some, it is clear that the riparian spaces are not safe, or to be enjoyed, but to be feared. Instances of crime or violence, although rare, fuel the idea that riparian spaces are unsafe and should be avoided. They become urban legends, told and retold. The rivers may have held meaning in the past but now some interviewees are wary of accessing these spaces. This feeling of uncertainty around these riparian spaces may in fact encourage young people to congregate there as they are less likely to be observed by parents, neighbors, or relatives who might see their “free” behavior as immoral in what is still a relatively conservative Christian context. We observed a group of young people (Figure 15) enjoying time with their girlfriends and boyfriends and making TikTok videos. This would be difficult to do in their home neighborhood, and this space allowed them freedom of expression.

So for some, insecurity is a reason not to visit the rivers, but for others, it is a way to claim ownership of these spaces and themselves. Other younger children are free to remove clothing and jump in the water, enjoying swimming with their friends, and teaching each other to dive. There is a small amount of literature on the importance of urban rivers for social connectivity (41). We also spoke to a homeless man for whom the rivers and the riparian land were a vital part of his daily life, helping him to have dignity, to wash his body and his clothing, and to relax away from the hectic life of a street sleeper. The importance of urban riparian and green spaces for homeless people has been well-documented (42, 43) and his proprietary attitude to the rivers was heartening, since few people felt strongly that they had positive feelings about the rivers as they currently are. Since homeless people are on the margins of society, and excluded from much social life, the rivers become a conduit to humanity and liminal landscapes such as these are vital to homeless peoples' survival.

One interesting idea raised by several interviewees is the idea that the mixing of tribes or the “dilution” of cultural norms was responsible for the environmental destruction which we now see. The idea is, as another interviewee put it, a “good argument for tribalism.” However, is this merely an excuse, a form of xenophobia, to blame others for the state of these rivers today? There is some evidence in the literature that cultural dilution does indeed lead to cultural erosion and the undermining of taboos which in the past supported environmental maintenance (44), and indeed that cultural dilution is reported to lead to social problems (45) in addition to environmental ones.

Riparian spaces are dichotomous: for some, a sanctuary and an oasis amid urban chaos; and for others, a dangerous, filthy hazardous place to be avoided. I, therefore, contend that urban rivers are liminal landscapes (46), an in-between place that is not one thing nor another, with no firm translation or characterization, and one for which use and interaction are contested.

### 4.3. One Health

The Mbagathi, Kandisi, and Kiserian Rivers present a quintessentially One Health challenge: wildlife, livestock, and an enormous surge in human population come together putting exponential pressure on a fragile ecosystem and environment. As one interviewee said, “Rongai *is* the rivers.” Indeed, Ongata Rongai would not exist without these rivers bounding it. They were one reason for the initial settlement of the area, but people now report cholera and other waterborne diseases as common, with much of this blamed on the rivers.

Without healthy rivers, there will be a dangerous, knock-on effect on human and animal health. Livestock reportedly refuse to drink from the rivers on occasion because of the smell and the taste. Yet, rivers are integral to healthy people and healthy animals. The spread of antimicrobial resistance, another quintessentially One Health topic, is exacerbated by poor riparian health in locations globally: “Discharge of untreated sewage into the rivers is a another major factor contaminating the river and facilitating the spread of AMR” (47). Anthropogenic activities around riparian spaces are known to contribute to the spread of antimicrobial resistance (48). I cannot claim with certainty that AMR, a quintessentially One Health problem, is occurring here, since I do not have the microbiological data to support this. However, we know that in the existing literature globally, this is occurring in rivers and riparian environments. A One Health approach to urban rivers is essential to ensure the health and wellbeing of all who use them. The findings of this study call for further microbiological research on AMR in these rivers and the users of the rivers.

### 4.4. The importance of ethnography: why a mixed methods anthropological approach to a One Health problem is important

The multiple methods and ethnographic approach to this study allowed for the triangulation of findings, supporting lengthy narratives with photographic evidence of how Rongai has changed over time, alongside archival reports and maps. Without these multiple data sources, it would not have been possible to create such an in-depth contextualization of the narrative interviews.

The meanings people ascribe to places such as rivers tell us a lot about how they perceive that environment, and how they use or misuse it. If these places have lost meaning culturally or socially, there will be little motivation to preserve this environment. For the middle-class Kenyan living in Rongai, the existence of the rivers is peripheral to their daily activities. The presence, however, of trash in their car on their way to work is much more present, so they may not think twice about throwing an empty plastic bag out of the window as they cross the bridge over the river. This plastic bag ends up floating in the river, possibly being consumed by a cow whose herder then must deal with the consequences to their cow's health. The many issues presented in this article show the struggles of river users and the loss of meaning of riparian spaces.

This disconnect among human social classes, livestock, and their environment disrupts the Complex Systems Theory approach—that One Health is only achievable (and by that, I mean health for all actors within the One Health system) through the functionality of these systems: human, animal, and environmental wellbeing working together for wellness. However, one must also acknowledge the complexity of these inequalities within One Health, and the various intersections of class, gender, and marginalized economic activities. An updated version of Systems Theory, called Complexity Theory or Complex Adaptive Systems Framework, goes some way to addressing these intersections and the complex intersections of class and gender while moving away from reductionist labeling of categories (21). This theory helps us to frame the findings here, that people and animals are constantly adapting to the changing and rapidly degrading environment in which they find themselves, and the dynamics between each element.

There is a Kiswahili saying: *raha ya dunia ni maelewano* (the happiness of the world is found in understanding [each other]). Without the approach I have used here, that of the ethnographic encounter and a deep understanding of the cultural shift in the meaning of riparian spaces, as well as the importance or unimportance of them to various demographics, it is impossible to fully understand the significance of these systems of One Health and wellbeing for all who use the riparian environment, and the environment itself which is perhaps the most neglected of the three tenets of One Health.

## 5. Conclusion

This study of the urban rivers in Ongata Rongai in Kenya found that rapid population growth and urbanization have placed huge pressure on this riparian environment, leading to significant environmental degradation with cumulative effects on humans and animals. Sewage is a particular menace cited by all interviewees, which affects businesses, individuals, livestock, and wild animals alike. People are usually aware of the dangers of river water, yet with unreliable access to water within their homes or neighborhoods (20), they have little alternative and must depend on this dangerous source of water.

Narratives were often dystopian in nature, especially with regard to the future of the Rongai environment, and reflected a global theme of negligence toward climate-related issues at a state level. In addition to this moribund attitude toward environmental destruction, there was an understanding that cultural change (largely blamed on a combination of recent cultural diversification in Rongai, and the proliferation of evangelical Christianity to the detriment of indigenous belief systems) has led to a laissez-faire approach to the environment in Rongai, whereas the riparian zones once held great meaning and functionality for the two or three cultural groups living in the area. Indigenous belief systems and prohibitions were formerly responsible for environmental preservation but these no longer exist, having been branded “devilry” by the (white) missionaries.

The rivers have evolved from being a place of prayer and medicine-gathering to a dangerous place where women are raped and muggings occur, as well as being a last bastion for “dangerous” wild animals. People and animals can no longer share space, but animals must be chased away or even killed. Yet, for young people, riparian forest is one place where they can explore, push boundaries, and experience some freedom from social norms.

Let us be clear: if the relevant government agencies remain stagnant on these issues, many more people will die from preventable waterborne diseases, livestock will die, wildlife will die, and the environment may be beyond redemption. This is a dystopian but very real vision of the future for Rongai, as envisioned by study participants. The rivers will be unusable, and a hazard if nothing is done, they reported. This is not a problem specific to Ongata Rongai but applies to all of Nairobi's rivers, and indeed urban rivers globally. This study merely serves as a snapshot of these spaces, which seems to be likely will be under increased population pressure if urban spaces continue to grow. Indeed, our president Ruto only in the past weeks raised the issue of the deplorable state of urban riparian spaces (49). But will this just be more talk, or will it lead to action?

The problems of these urban rivers in Ongata Rongai cannot be overstated: this study finds that humans and animals are reliant on these riparian environments, and rivers are often the reason for settlement of these spaces historically, but today the rivers are no longer an asset but a hazard for all users of these spaces: narratives showed that women are raped, robberies occur, animals and humans drink poisoned water, crops shrivel and die, and fish die or swim on their sides, poisoned by the river water. The state of these rivers is surely an ecological disaster for all.

Yet, others still find value in these spaces: young people experience relative freedom here to hang out with friends and lovers, homeless people gain respite from the drudgery of daily survival, and despite the dangers for health, farmers, fishers, and livestock owners seek a living. There is, for many, no alternative.

As a resident of Ongata Rongai, I felt exasperated by the lack of structural-level action on issues that are in plain sight. These are not hidden problems, or issues affecting only a proportion of the population. Riparian health affects everyone and has a knock-on effect on water safety, food safety, and other diverse factors.

Several important issues were raised during this study which are important for future study. The prevalence of diarrhea and related diseases in the study site is unknown but often cited by participants during interviews for this study. It is also important to understand peoples' awareness of microbial contamination, AMR, and the links between human and animal health and diseases, since my study touched on this but further investigation would be prudent. Furthermore, microbiological samples of the river water, riverbed sediment, and riverbank farm soil would be crucial in understanding the true extent of AMR pathogens in Ongata Rongai. Some of the narratives presented here touched on the issue of climate change and disasters, but an in-depth study into urban rivers and climate change would shed light on the extent of the impact of these on local populations.

There are clear policy implications for this study. The most obvious seems to be the fair and comprehensive enforcement of existing legislation regarding sewage and solid waste disposal. SDG6, clean water, and sanitation, calls for this basic provision for humans globally. However, as demonstrated by this research, this is far from a realized goal in Ongata Rongai. Access to clean water is not equitable, nor is it universal. By the rivers, livestock owners, laundry people, and swimmers contend with raw sewage and trash: SDG 6.3 specifically aims to address this, and significant financial and structural inputs will be required to tackle this by 2030. Indeed, SDG 6.6 (“protect and restore water-rated ecosystems”) seems a long way from realization given the current state of degradation described and observed in Ongata Rongai.

The county government needs to be aware, if they are not already, of the implications for human and animal health if the current situation were to continue. Given that Ongata Rongai is now part of a municipality, it has the power to make these policy changes at a community level. Polluters of the river and riparian spaces must be prosecuted regardless of who they are. Additionally, Nairobi Municipality needs to provide a local sewage treatment plant, as well as extensive recycling and solid waste disposal services. Many of the issues faced by river users are only able to be tackled at a policy level, although changes in individual- and community-level understanding of the importance of urban rivers would also be necessary. Community sensitization and workshops, as well as provision of plastic alternatives (for example, encouraging the use of reusable and washable diapers), at a community level are needed, and sites for disposal of trash are accessible to all.

The time for talking is past. Now we must have action if we are to ensure the health and wellbeing of humans and animals who depend on urban riparian spaces globally.

## Data availability statement

The raw data supporting the conclusions of this article will be made available by the authors, without undue reservation.

## Ethics statement

The studies involving human participants were reviewed and approved by ILRI Research Ethics Committee (approval number ILRI-IREC2021-09). Written informed consent for participation was not required for this study in accordance with the national legislation and the institutional requirements.

## Author contributions

OH conceived the study, conducted fieldwork, analyzed the data, and wrote the manuscript.

## Footnotes

^1^Sustainable Development Goals, United Nations.

^2^The two are used interchangeably here in the paper and in the community.

^3^The Kenya Wildlife Service.

^4^Kware is an alternative pronunciation of quarry.

^5^Kiswahili, meaning farm.
